# Exploring natural genetic diversity in a bread wheat multi-founder population: dual imaging of photosynthesis and stomatal kinetics

**DOI:** 10.1093/jxb/erae233

**Published:** 2024-05-25

**Authors:** Michele Faralli, Greg Mellers, Shellie Wall, Silvere Vialet-Chabrand, Guillaume Forget, Alexander Galle, Jeron Van Rie, Keith A Gardner, Eric S Ober, James Cockram, Tracy Lawson

**Affiliations:** School of Life Sciences, University of Essex, Colchester CO4 3SQ, UK; NIAB, 93 Lawrence Weaver Road, Cambridge CB3 0LE, UK; School of Life Sciences, University of Essex, Colchester CO4 3SQ, UK; School of Life Sciences, University of Essex, Colchester CO4 3SQ, UK; University of Bordeaux, INRAE, UMR BIOGECO, Pessac 33615, France; BASF Belgium Coordination Center CommV-Innovation Center Gent, Technologiepark-Zwijnaarde 101, 9052 Gent, Belgium; BASF Belgium Coordination Center CommV-Innovation Center Gent, Technologiepark-Zwijnaarde 101, 9052 Gent, Belgium; NIAB, 93 Lawrence Weaver Road, Cambridge CB3 0LE, UK; International Maize and Wheat Improvement Center (CIMMYT), Carretera México-Veracruz, Mexico; NIAB, 93 Lawrence Weaver Road, Cambridge CB3 0LE, UK; NIAB, 93 Lawrence Weaver Road, Cambridge CB3 0LE, UK; School of Life Sciences, University of Essex, Colchester CO4 3SQ, UK; Lancaster University, UK

**Keywords:** Kinetics, MAGIC, photosynthesis, photosynthetic capacity, stomatal conductance, thermal images, water use efficiency, wheat

## Abstract

Recent research has shown that optimizing photosynthetic and stomatal traits holds promise for improved crop performance. However, standard phenotyping tools such as gas exchange systems have limited throughput. In this work, a novel approach based on a bespoke gas exchange chamber allowing combined measurement of the quantum yield of PSII (*F*_q_'*/F*_m_'), with an estimation of stomatal conductance via thermal imaging was used to phenotype a range of bread wheat (*Triticum aestivum* L.) genotypes. Using the dual-imaging methods and traditional approaches, we found broad and significant variation in key traits, including photosynthetic CO_2_ uptake at saturating light and ambient CO_2_ concentration (*A*_sat_), photosynthetic CO_2_ uptake at saturating light and elevated CO_2_ concentration (*A*_max_), the maximum velocity of Rubisco for carboxylation (*V*_cmax_), time for stomatal opening (*K*_i_), and leaf evaporative cooling. Anatomical analysis revealed significant variation in flag leaf adaxial stomatal density. Associations between traits highlighted significant relationships between leaf evaporative cooling, leaf stomatal conductance, and *F*_q_'*/F*_m_', highlighting the importance of stomatal conductance and stomatal rapidity in maintaining optimal leaf temperature for photosynthesis in wheat. Additionally, *g*_smin_ and *g*_smax_ were positively associated, indicating that potential combinations of preferable traits (i.e. inherently high *g*_smax_, low *K*_i_, and maintained leaf evaporative cooling) are present in wheat. This work highlights the effectiveness of thermal imaging in screening dynamic *g*_s_ in a panel of wheat genotypes. The wide phenotypic variation observed suggested the presence of exploitable genetic variability in bread wheat for dynamic stomatal conductance traits and photosynthetic capacity for targeted optimization within future breeding programmes.

## Teaser

Significant variation exists in stomatal dynamics and photosynthetic capacity in a MAGIC bread wheat population.

## Introduction

Crop yield is the product of the cumulative rates of photosynthesis over the growing season (Zelitch, 1982). Indeed, several developmental processes occurring throughout the life cycle of a crop co-determine a series of yield components that are often limited by the availability of assimilates (Slafer, 2003). For instance, free-air concentration enrichment (FACE) experiments (Long *et al*., 2006) and bioengineering approaches (Driever *et al.*, 2017) have provided evidence that increasing rates of photosynthesis can lead to yield gains. In many crops, while harvest index and light interception capacity are approaching their theoretical maximum (∼0.64 and 0.8–0.9, respectively, Long *et al*., 2006), the efficiency of energy conversion into biomass (i.e. radiation use efficiency and thus photosynthesis) still has substantial room for improvement (Long *et al*., 2006). Although it is well established that significant variation in photosynthesis exists between species (e.g. Wullschleger, 1993; Lawson *et al.*, 2012), several more recent studies have reported significant variation between cultivars of the same species (Driever *et al*., 2014; Carmo-Silva *et al*., 2017; Faralli *et al*., 2019a, b; Ferguson *et al*., 2020; McAusland *et al.*, 2020; Wall *et al.*, 2023). Most of the intraspecific natural variation in photosynthesis for C_3_ plants has been attributed to differences in biochemical capacity, including electron transport rates and carboxylation efficiency (Driever *et al*., 2014; Carmo-Silva *et al*., 2017). In addition, under natural dynamic conditions, photosynthetic processes such as activation of Calvin cycle enzymes and/or stomatal dynamics can also be limiting (Lawson and Blatt, 2014; Taylor and Long, 2017; Faralli *et al*., 2019a; Salter *et al*., 2019).

Stomatal dynamics balance leaf CO_2_ uptake and water loss, and hence significantly influence the key components of crop productivity: the cumulative rate of photosynthesis, water use, and evaporative cooling. The opening and closing of stomata are driven by a series of environmental, hormonal, and hydraulic signals (Blatt, 2000), with significant variation observed in sensitivity and responsiveness among different species (Lawson *et al.*, 1998, 2003, 2012; Lawson, 2009) and genotypes driven by differences in morphology (Weyers and Lawson, 1997; Weyers *et al*., 1997; Hetherington and Woodward, 2003; Drake *et al*., 2013; McAusland *et al.*, 2015; Yoshiyama *et al*., 2024). In general, stomata open in response to increasing light intensity, low CO_2_ concentration ([CO_2_]), high temperatures, and low vapour pressure deficit (VPD), while closure is driven by low light or darkness, high [CO_2_], and high VPD (Outlaw, 2003). In the natural environment, these factors can occur simultaneously and in a dynamic and fluctuating manner (Sharkey and Raschke, 1981; Zeiger and Zhu, 1998; Talbott *et al*., 2003).

It has been extensively shown that the rapidity in stomatal movements under conditions such as shade or sun-flecks may be considered preferable traits for crop improvement due to reduced water loss when carbon gain is limited and a reduction in diffusional constraint on photosynthesis (Lawson *et al.*, 2010; Lawson and Blatt, 2014). In wheat (*Triticum aestivum* L.), a major food crop accounting for up to 20% of the world’s calorie consumption (Erenstein *et al*., 2022), these traits have been sparsely explored (Faralli *et al*., 2019a), although in other crops (e.g. rice) stomatal rapidity has been associated with an adaptation to dry conditions (Qu *et al*., 2016; Faralli *et al*., 2019a; Zhang *et al*., 2019). Therefore, the main objective of this work was to phenotype, via a novel, non-invasive, and high-throughput method, stomatal rapidity in a wheat multi-parent advanced generation intercross (MAGIC) population along with steady-state gas exchange traits. A total of 192 greenhouse-grown wheat genotypes were analysed, including photosynthetic capacity and stomatal traits. Our work is the largest phenotyping study carried out so far to characterize wheat stomatal responses and photosynthetic diversity under dynamic light, and may provide methods and resources to open up new avenues to optimize wheat responses to natural fluctuating environmental conditions.

## Materials and methods

### Plant material

The MAGIC wheat population was used in this study (Mackay *et al.*, 2014). The population consists of recombinant inbred lines (RILs) generated from three cycles of intercrossing between eight elite European wheat cultivars (Alchemy, Brompton, Claire, Hereward, Rialto, Robigus, Soissons, and Xi-19) followed by five rounds of self-pollination to derive RILs as described by Mackay *et al.* (2014). A subset of the population comprising 192 lines and the eight parental lines was used for this work. These lines encompassed the important genetic variation present in the larger population.

### Plant growth, vernalization, and experimental design

Seeds were sown in plastic trays containing compost and germinated in a growth cabinet (Reftech BV, Sassenheim, the Netherlands) at the University of Essex at ~200 µmol m^–2^ s^–1^ photosynthetic photon flux density (PPFD), 14 h/10 h photoperiod (light/dark), ~15 °C on average, and ~60% relative humidity. The compost (Levington F2S; Everris, Ipswich, UK) contained coir, sand, and fertilizer (144 mg l^–1^ N, 73 mg l^–1^ P, 239 mg l^–1^ K, adjusted to pH 5.3–6.0 with dolomitic lime). At BBCH (Biologische Bundesanstalt, Bundessortenamt und Chemische Industrie) growth stage (GS) 12 (GS12, two seedling leaves unfolded; Lancashire *et al.*, 1991), seedlings were moved into a cold room for vernalization: 4 °C, ~50 µmol m^–2^ s^–1^ PPFD at 10 h/14 h photoperiod (light/dark) for 8 weeks. After vernalization, seedlings (one per pot) were transplanted into 1.5 litre pots (15 cm diameter; 12 cm deep) containing F2S compost and transferred to a temperature-controlled glasshouse. To phenotype the population at flag leaf emergence (GS39–GS41), three batches of plants were grown from July 2017 to April 2018. Batch 1 consisted of 189 genotypes, with each line replicated twice. In Batch 2, 186 of these genotypes were grown again (*n*=2), but the eight parental lines had a higher number of replications (*n*=5). Batch 3 included an extra replicate of contrasting lines from Batch 1 (48 genotypes) and Batch 2 (47 genotypes), as well as 80 extra genotypes in *n*=2, along with 8-fold (*n*=8) parental replication. After vernalization, plants from each batch were transferred to the glasshouse and spatially randomized with a two-block structure, and genotypes exhibiting a non-uniform behaviour between replicates were discarded from the study (Supplementary Fig. S1).

### Phenotypic analysis

Plants were scored for the occurrence of flag leaf emergence (GS39) and flag leaf fully emerged (GS41). Plant height (soil surface to flag leaf tip) at GS41 was assessed with a ruler prior to stomatal conductance analysis. All measurements were made on flag leaves of plants that had reached GS41–GS45.

### High-throughput phenotyping of dynamic *g*_s_ responses with thermal imaging

#### Thermal imaging

Dynamic responses of stomatal conductance to water vapour (*g*_s_) to a step change in irradiance were assessed by modifying an in-house system developed at the University of Essex (McAusland *et al.*, 2013). A FluorImager system (Technologica, Colchester, Essex, UK) was modified to allow thermal imaging and chlorophyll fluorescence imaging simultaneously. For thermal imaging, a thermal camera Optris 450i (Optris GmbH, Berlin, Germany) with a temperature resolution of 0.1 °C was configured with an emissivity (ε) of 0.96 and set to perform a non-uniform calibration every minute. The thermal camera was positioned at 0.45 m in the original location of the chlorophyll fluorescence camera, directly above the imaging port, while the chlorophyll fluorescence camera AVT manta (Allied Vision, Stadtroda, Germany) was positioned at a 90° angle. A silver-coated mirror (Thor-Optics, Dachau, Germany) was hinged on an axis directly above the original camera port and a servo motor connected to an Arduino was used to automatically move the mirror, switching between the two cameras while keeping the same field of view (Supplementary Fig. S1). Air temperature and relative humidity were collected using a sensor (HygroClip2, HC2A-S, Rotronic, Bassersdorf, Switzerland) connected to the same Arduino. A custom software was used to automatically collect images from both cameras, record environmental data, operate the servo motor, and process the data.

#### Imaging chamber and gas control

A modified open-top chamber based on that described by McAusland *et al.* (2013) was designed and constructed. The cuvette allowed the control of the concentration of gases (N, H_2_O, CO_2_, and O_2_), while keeping an open top for imaging. The chamber was built from Perspex and, due to the relatively high reflection inside the chamber at high light intensity, the interior surfaces were painted grey. With the exception of the base, the chamber consisted of an inner and outer wall separated by a 10 mm gap. The outer walls were connected on each of the four sides by 6 mm PTFE tubing connections that fed gas into the chamber wall cavity. The inner wall was perforated with 1 mm diameter holes at a density of 9 per 100 mm^2^, which was optimal for maintaining homogenous gas concentrations whilst minimizing leaf movement through turbulence. Within the chamber, target gas concentrations of nitrogen (N_2_) and CO_2_ were individually maintained by mass flow controllers (EL Flow, Bronkhorst, Ruurlo, the Netherlands), connected to compressed gas cylinders containing 100% N_2_ and CO_2_, respectively (British Oxygen Company-Industrial Gases, Ipswich, UK). To control water vapour concentration, a Controlled Evaporation and Mixing system (CEM Evaporator W-202A, Bronkhorst, Newmarket, UK) was used to precisely regulate the water vapour content of the air. Gas composition in the chamber was monitored at leaf height by sampling air with a diaphragm pump (Type 124, ADC Hoddesdon, Herts, UK) at 500 cm^3^ min^–1^. Both CO_2_ and H_2_O vapour concentrations were measured with an infrared gas analyser (IRGA) (Li- 840, LI-COR, Lincoln, NE, USA). Throughout the phenotyping experiment and during each analysis, ambient CO_2_ concentration (*C*_a_) was maintained at 400 µmol CO_2_ mol^–1^ air while relative humidity was maintained at 45–60% inside the cuvette.

### Estimating dynamic stomatal conductance from leaf temperature

Significant negative correlation exists between leaf conductance to water vapour and leaf temperature (Jones *et al.*, 2009). Due to the transition of water into water vapour during transpiration, energy (latent heat of vaporization) is taken from the leaf, leading to a reduction in surface leaf temperature. This evaporative cooling effect of transpiration can be used as an indirect method for the estimation for leaf *g*_s_ (Supplementary Fig. S1). For this reason, *g*_s_ was estimated following the equations proposed by Leinonen *et al*. (2006) and shown in Jones *et al.* (2009).

Mass leaf transpiration was estimated as follows:


$$\begin{array}{l} E_{m}=\frac{\left[0.92g_{b}+\left(\frac{4\varepsilon \sigma T_{a}^{3}}{\rho C_{p}}\;\right)\right]\left(\rho C_{p}\right)\left(T_{dry}-T_{leaf}\right)}{\lambda } \end{array}$$

Where *g*_b_ is the estimated boundary layer conductance to water vapour (see Supplementary Dataset S1 for an example of calculation), 0.92 indicates a proportional relationship between heat and water vapour transfer rates across the boundary layer under laminar flow, ε represents sample emissivity, σ is the Stefan–Boltzmann constant, *T*_a_ is air temperature, ρ represents air density, *C*_p_ is the air specific heat capacity, *T*_dry_ is the temperature of the dry reference, *T*_leaf_ is the temperature of the leaf sample, and λ is the latent heat of vaporization.

Conversion of *E*_m_ to mol m^–2^ s^–1^ was carried out and total conductance to water vapour was estimated as:


$$\begin{array}{l} g_{w}=\frac{E}{\frac{e_{s}-e_{a}}{P}} \end{array}$$

where *E* is leaf transpiration, *e*_s_ is the saturated water vapour pressure in the leaf, *e*_a_ is the air vapour pressure, and *P* is the atmospheric pressure (i.e. leaf VPD).

Leaf stomatal conductance to water vapour (*g*_s_) was then estimated as


$$\begin{array}{l} g_{s}=\frac{1}{\frac{1}{g_{w}}-\frac{1}{g_{b}}} \end{array}$$

A spreadsheet containing an example of calculating *g*_s_ from thermography and environmental conditions is provided in Supplementary Dataset S2.

### Protocol for estimating parameters in response to step changes in irradiance

Prior to each analysis, plants were moved from the greenhouse to a controlled-environment dark room (20 °C maintained with an air conditioner and ~60% relatuive humidity maintained with a humidifier). It was possible to run three plants simultaneously together with the dry reference. The dry reference was a flag leaf sampled with scissors from spare plants for each protocol, and petroleum jelly (Vaseline) was applied on both surfaces to prevent transpiration. Dark-adapted plants (~1 h per cycle, see below) were clamped onto the chamber and acclimated for 16 min at 100 µmol m^–2^ s^–1^ PPFD followed by a step change in light to 1000 µmol m^–2^ s^–1^ PPFD for 30 min (sufficient to reach steady state in wheat, Faralli *et al*., 2019a), and thermal and fluorescence images were taken every 2 min. The thermal dataset generated was automatically analysed with a bespoke system based on OpenCV that estimates leaf *g*_s_ in a pixel-based manner and averages over the leaf sample. Leaf segmentation was carried out using basal fluorescence in the dark and by applying Otsu thresholding. The time constant for the rapidity of *g*_s_ response to a step change in light intensity (*K*_gs_, estimated from *g*_s_ kinetics; or *K*_t_, estimated from *T*_dry_–*T*_leaf_ kinetics) was estimated with the model proposed by Vialet-Chabrand *et al.* (2013). The dynamic model predicts the temporal response of *g*_s_ at the leaf level using a sigmoid function for increasing *g*_s_. It describes the temporal response of *g*_s_ using a time constant (*k*, min), an initial time lag (λ, min), and a steady-state *g*_s_ reached at a given PPFD. The model also allowed the estimation of *g*_s_ at 100 µmol m^–2^ s^–1^ PPFD (*g*_smin_) and at 1000 µmol m^–2^ s^–1^ PPFD (*g*_smax_). In addition, Δ*T* was estimated as the difference in temperature between the first point after the step change in light and the last point (i.e. cooling capacity). The light-adapted quantum yields of PSII (*F*_q_'/*F*_m_') at low and high light were also assessed as well as non-photochemical quenching (NPQ: high light) estimated following Murchie and Lawson (2013).

### Photosynthetic CO_2_ response curves (*A*/*C*i)

The same leaf used for imaging was subjected to gas exchange measurements. Photosynthesis measurements (*A*/*C*_i_ curves) were performed between 09.00 h and 15.00 h on the fully emerged flag leaf at GS41–GS45 using a LI-6400. Measurements of the response of *A* to substomatal CO_2_ concentrations (*C*_i_) were performed in the middle of the tagged leaf using an open infrared gas exchange system and a 2 cm^2^ leaf cuvette with an integral blue–red LED light source (LI-6400-40; LI-COR). In the cuvette, PPFD was maintained at a saturating level of 1500 µmol m^–2^ s^–1^, a leaf temperature of 20 ± 0.1 °C, a VPD between 0.9 kPa and 1.3 kPa, and a *C*_a_ of 400 µmol mol^–1^. When steady-state conditions were achieved, *C*_a_ was sequentially decreased to 300, 200, 100, and 75 µmol mol^–1^ before returning to the initial concentration of 400 µmol mol^–1^. This was followed by a sequential increase to 550, 700, 1000, and 1200 µmol mol^−1^. Readings were recorded when *A* had stabilized to the new conditions. The maximum velocity of Rubisco for carboxylation (*V*_cmax_) and the maximum rate of electron transport demand for ribulose 1,5-bisphosphate (RuBP) regeneration (*J*_max_) were derived by curve fitting. We used the Plantecophys R package to determine *V*_cmax_ and *J*_max_ via non-linear least squares, while standard errors of the parameters were estimated with standard methods (Supplementary Fig. S1).

### Validation of the estimated *g*_s_ dataset with thermal imaging

An additional experiment was conducted to validate image-based estimations of *g*_s_ by comparison with *g*_s_ values estimated using standard gas exchange measurements. Lines with contrasting *K*_gs_ values were grown as for the phenotyping experiment. IRGAs (Li-Cor 6400XT) assessed the rapidity of stomatal responses to a step change in light (using the same protocols as described by Faralli *et al*., 2019a). Briefly, prior to measurement, flag leaves of plants at GS41 were equilibrated to a PPFD of 100 µmol m^–2^ s^–1^ for ~60 min or until *g*_s_ reached ‘steady state’, defined as a ≤2% change in rate during a 10 min period. After equilibration, PPFD was increased to 1500 µmol m^–2^ s^–1^ for 50 min and subsequently returned to 100 µmol m^–2^ s^–1^ for 1 h. Conditions inside the leaf cuvette were kept constant at 20 ± 0.1°C leaf temperature, a VPD of 1 kPa with a dew point generator (LI-610; LI-COR), and 400 µmol CO_2_ mol^–1^ air (ambient CO_2_ concentration, *C*_a_). The time constant for the rapidity of *g*_s_ responses to a step change in light intensity was estimated as previously described.

### Statistical analysis

Data analysis was conducted using Rstudio. Due to significant variation in growing conditions in the glass house and spatial effects, the data were analysed using a one-way analysis of covariance (ANCOVA), treating ‘greenhouse’ and ‘block’ as covariates. Normality checks were performed on all datasets. The associations between different traits were examined through correlation analysis, and the Pearson test was used to evaluate the strength of these associations.

## Results

### Comparison of *g*_s_ values obtained from imaging and standard approaches

**Fig. 1.**
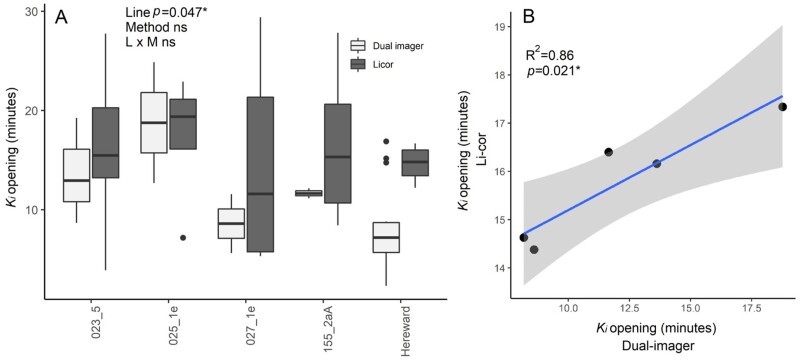
Comparison of methods for the measurement of stomatal opening. (A) Time constant for stomatal opening analysed via an infrared gas analyser (dark grey) and the dual imager (light grey) (*n*=3–7) in a subset of wheat genotypes. Data were analysed with two-way ANOVA and significant differences are indicated. (B) The linear association between the two methods. Data are means, and fitting was carried out via linear regression.

To evaluate *g*_s_ values obtained from the dual imaging system compared with those from standard IRGA methods, a comparative analysis was performed on a subset of genotypes, selected based on their differential kinetic profiles (Fig. 1). Overall, the time constant for stomatal opening (*K*_i_) as determined by the dual imager method and standard gas exchange showed no significant difference between the two methods (Fig. 1A) (*P*>0.05). Additionally, a positive and significant association was observed for dual imager and IRGA estimates of *K*_i_ (Fig. 1B). Generally, the dual imager method showed higher variation between genotypes compared with the IRGA, yet the ranking among lines remained consistent.

### Photosynthetic capacity

**Fig. 2.**
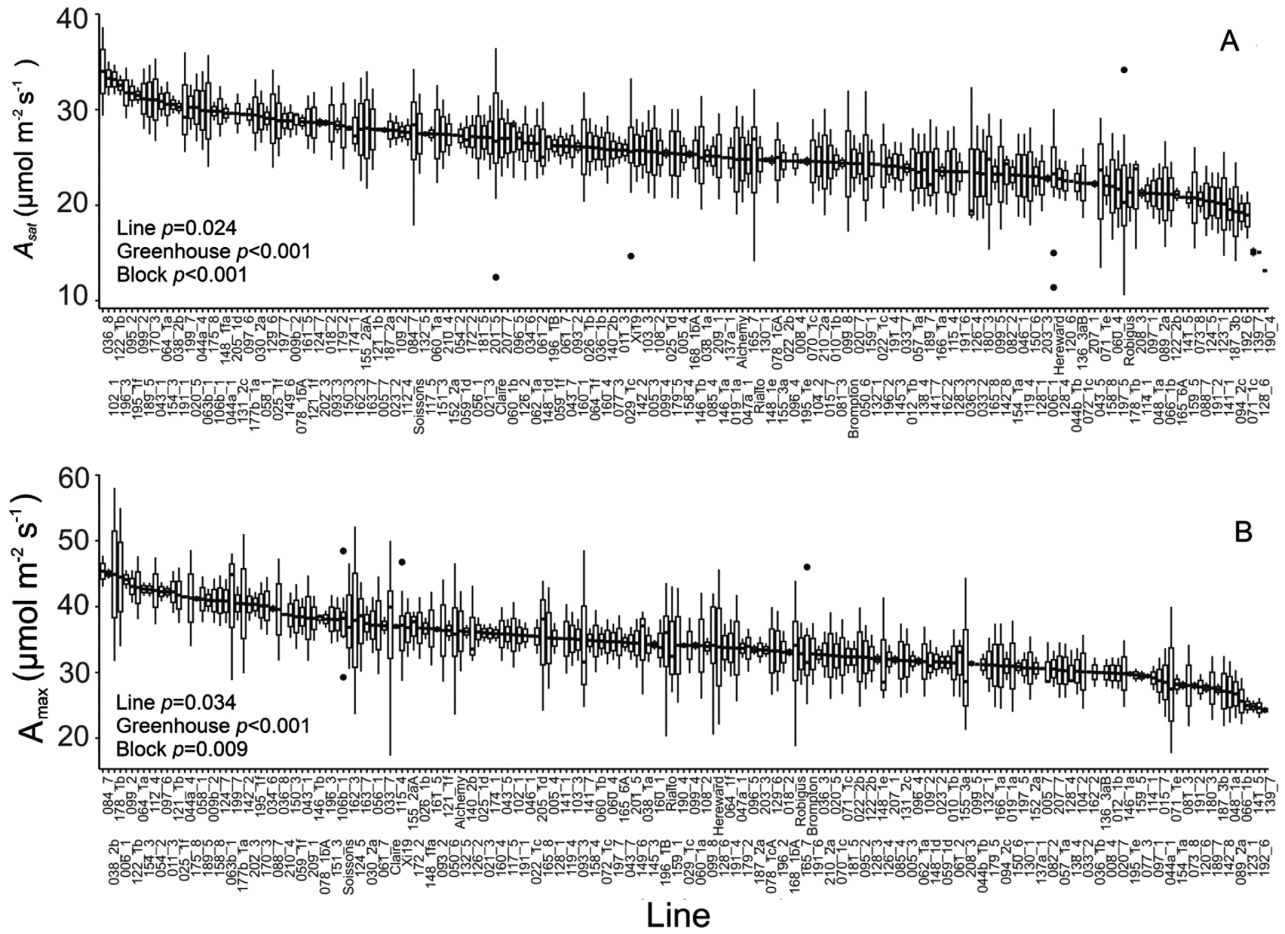
Steady-state photosynthetic traits estimated via *A*/*C*_i_ curves. (A) The variation between lines for photosynthetic CO_2_ uptake at saturating light and ambient CO_2_ concentration (*A*_sat_). (B) The variation between lines for photosynthetic CO_2_ uptake at saturating light and elevated CO_2_ concentration (*A*_max_). For graphs, horizontal lines within boxes indicate the median and boxes indicate the upper (75%) and lower (25%) quartiles. Whiskers indicate the ranges of the minimum and maximum values. Circles indicate outliers. Data were analysed with ANCOVA (*n*=2–10), and *P*-values for the main effects are shown in the graph.

The response of the photosynthetic assimilation rate to increasing CO_2_ at saturating irradiance (*A*/*C*_i_ response curve) was analysed for all lines to assess variation in photosynthetic capacity. *A*/*C*_i_ curves for all genotypes measured followed the typical hyperbolic response. From these response curves, both the light-saturated rate of photosynthesis (*A*_sat_) and the light- and CO_2_-saturated rate of photosynthesis (*A*_max_) were determined. Significant differences (*P*<0.05, Fig. 2) were observed between genotypes in both values, indicating variation in the maximum photosynthetic potential as well as operational rate across these genotypes. *A*_sat_ varied from a minimum of 18 µmol m^–2^ s^–1^ to a maximum of 34 µmol m^–2^ s^–1^ on average. For the eight parental lines, ‘Hereward’ and ‘Robigus’ showed the lowest (20–21.5 µmol m^–2^ s^–1^) values, while the highest values (27–28 µmol m^–2^ s^–1^) were observed in ‘Soissons’, ‘Claire’, and ‘Xi19’. For RILs of the population, the highest values were observed for MEL_122_1b and MEL_036_8 (up to 34 µmol m^–2^ s^–1^), while the lowest values between 15 µmol m^–2^ s^–1^ and 18 µmol m^–2^ s^–1^ were observed in MEL_139_7 and MEL_071_1c. *A*_max_ varied significantly between genotypes, with values ranging from 25 µmol m^–2^ s^–1^ to 45 µmol m^–2^ s^–1^ on average, and a positive association between *A*_sat_ and *A*_max_ was observed (*P*<0.001). However, in some cases, the ranking was significantly altered; for example, Robigus had a relatively low *A*_sat_ while it was positioned in the middle of the distribution for *A*_max_. *A*_sat_ and *A*_max_ for the 200 lines measured followed a normal distribution. Therefore, the differential ranking of lines between operational assimilation rates (*A*_sat_) and maximum capacity (*A*_max_) clearly indicates that these are influenced by differing factors and that a high photosynthetic potential does not always translate to a higher actual rate in the field.

**Fig. 3.**
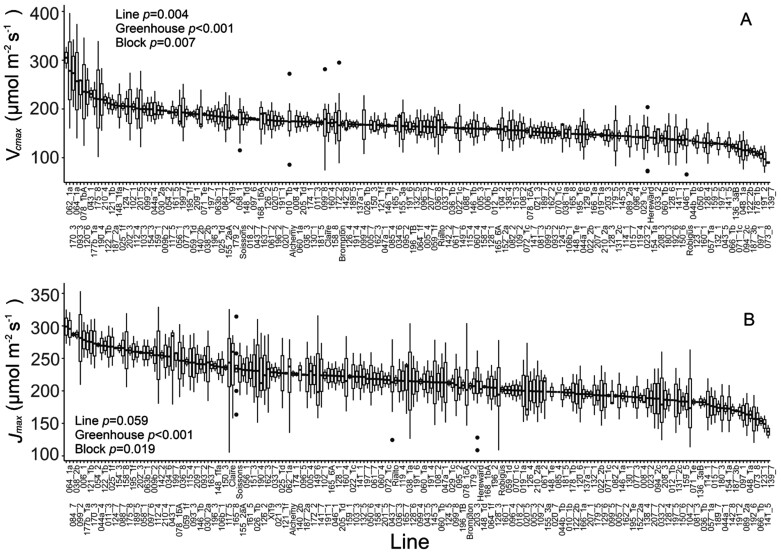
Biochemical traits estimated via *A*/*C*_i_ curves. (A) The variation between genotypes for the maximum velocity for Rubisco carboxylation (*V*_cmax_). (B) The variation between genotypes for the maximum electron transport rate for RuBP regeneration (*J*_max_). For graphs, horizontal lines within boxes indicate the median and boxes indicate the upper (75%) and lower (25%) quartiles. Whiskers indicate the ranges of the minimum and maximum values, and dots indicate outliers. Data were analysed with ANCOVA (*n*=2–10); *P*-values are indicated.

Significant variation in *V*_cmax_ was observed between genotypes (*P*<0.05. Fig. 3), with values varying >3-fold (from 90 µmol m^–2^ s^–1^ to 270 µmol m^–2^ s^–1^). As stated above, on average for parental lines, ‘Hereward’ showed the lowest (140 µmol m^–2^ s^–1^) values along with ‘Robigus’ (135 µmol m^–2^ s^–1^), while higher values were observed for ‘Xi19’ and ‘Soissons’ (180 µmol m^–2^ s^–1^). For RILs, MEL_139_7 and MEL_073_8 had the lowest values (~90 µmol m^–2^ s^–1^) while the highest values were observed for MEL_062_1a and MEL_170_3 (280–300 µmol m^–2^ s^–1^). The maximum electron transport rate capacity for RuBP regeneration (*J*_max_) also showed variation (although borderline significant, *P*=0.059) between genotypes, and ranged between 100 µmol m^–2^ s^–1^ and 310 µmol m^–2^ s^–1^ on average.

**Fig. 4.**
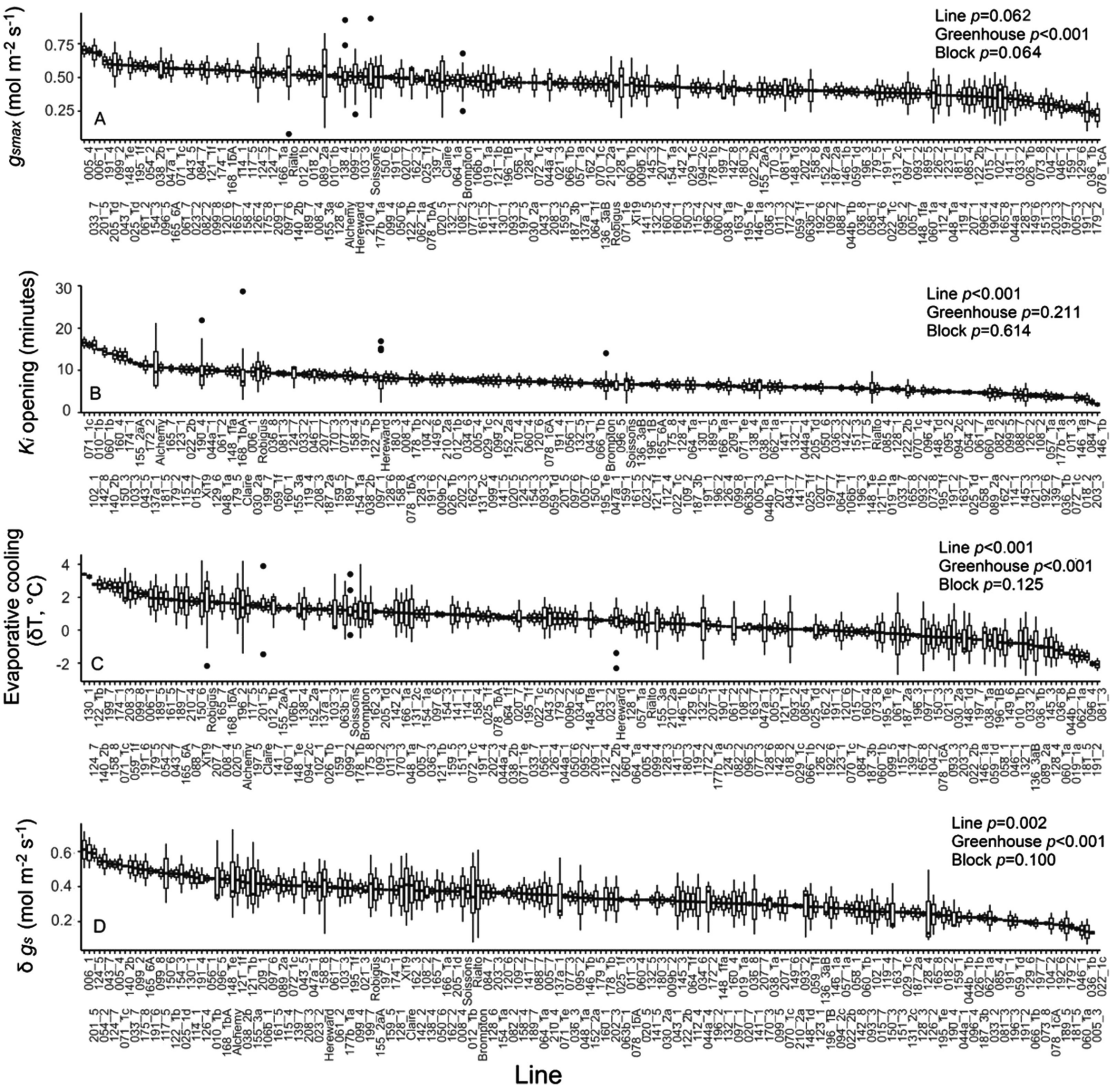
Maximum stomatal conductance under near-saturating light (*g*_smax_), time for stomatal opening after a step change in light (*K*_i_),evaporative cooling capacity (δ*T*), and the difference between maximum stomatal conductance under near-saturating light and stomatal conductance before the step change in light (δ*g*_s_) estimated with thermal imaging. For graphs, horizontal lines within boxes indicate the median and boxes indicate the upper (75%) and lower (25%) quartiles. Whiskers indicate the ranges of the minimum and maximum values. Data were analysed with ANCOVA (*n*=2–10), and *P*-values are shown in the graph.

In the field, the realized assimilation rate is influenced by *g*_s_ and the potential imposed diffusional constraints due to the dynamic behaviour of stomata. In order to assess stomatal dynamics in all 200 lines, a dual imager was utilized that adopted a high-resolution thermal camera to assess changes in leaf temperature as a proxy of *g*_s_ response (McAusland *et al.*, 2013; Vialet-Chabrand and Lawson, 2019, 2020) to a step increase in light intensity. Stomatal conductance values following 30 min at subsaturating light intensity (1000 µmol m^–2^ s^–1^) (*g*_smax_) showed trends of variation (*P*=0.064) between lines, and an average value of 0.7 mol m^–2^ s^–1^ (Fig. 4A). However, unsurprisingly, significant variation in individual measurements within the replicates was high (average SEM 0.1 mol m^–2^ s^–1^). In the parental lines, the lowest *g*_smax_ values (0.6 mol m^–2^ s^–1^) were observed in Robigus, while Rialto displayed the highest value (0.85 mol m^–2^ s^–1^). Several RILs had low *g*_smax_, ranging from 0.3 µmol m^–2^ s^–1^ to 0.5 mol m^–2^ s^–1^ (e.g. MEL_078_1cA and MEL_179_2). The rapidity of changes in *g*_s_ after a 30 min sun-fleck, measured as the time constant to reach 63% of *g*_smax_ (*K*_i_), varied greatly between lines (*P*<0.001), and values ranged between 1.9 min and 19 min (Fig. 4B). For the parental material, Rialto, Brompton, and Hereward had the fastest responses (5–8 min on average) while Xi19 and Alchemy showed the slowest response (10 min). MEL_146_1b and MEL_203_3 had a very quick stomatal response (*K*_i_ between 1.9 min and 2.5 min), while MEL_102_1 and MEL_071_1c had the slowest stomatal response (16 min). *K*_i_ values followed a normal distribution, with the majority of speeds of responses between 5 min and 13 min, which agrees with previous reports on wheat (Faralli *et al*., 2019a). Changes in stomatal conductance with differences in light intensity are critically important for evaporative cooling and maintaining optimal leaf temperature for photosynthesis and other metabolic processes. Differences between leaf temperature driven by the 30 min sun-fleck (i.e. cooling capacity, δ*T*) showed significant and wide variation with lines, and illustrate the ability of these lines to counteract the increase in irradiance via transpiration (i.e. positive evaporative cooling) (Fig. 4C). Values for δ*T* ranged between –2 °C and 4 °C changes in leaf temperatures. Values below zero indicate limited evaporative cooling with higher light intensity, whilst positive values show that changes in *g*_s_ result in increased cooling capacity. Most of the population (70%) showed positive values (i.e. cooling capacity) and all the parental material showed positive values, with Xi19, Robigus, and Alchemy showing the highest values (δ*T* 1.5 °C on average). For the parental lines, the lowest values were observed for Hereward and Rialto (δ*T* 0.5 °C) while MEL_081_3 and MEL_191_2 had the most negative values (δ*T* –2 °C). Significant differences were also observed for δ*g*_s_; that is, the change in stomatal conductance after a step change in light (Fig. 4D). In general, δ*g*_s_ ranged from 0.2 mol m^–2^ s^–1^ to up to 0.9 mol m^–2^ s^–1^, with Alchemy showing the highest values between parental lines (0.7 mol m^–2^ s^–1^) while Brompton and Rialto showed the lowest (0.5 mol m^–2^ s^–1^). MEL_201_5 and MEL_006_1 showed the highest delta between *g*_smin_ and *g*_smax_ (0.9 mol m^–2^ s^–1^) while MEL_005_3 and MEL_036_1b showed the lowest (0.2 mol m^–2^ s^–1^).

**Fig. 5.**
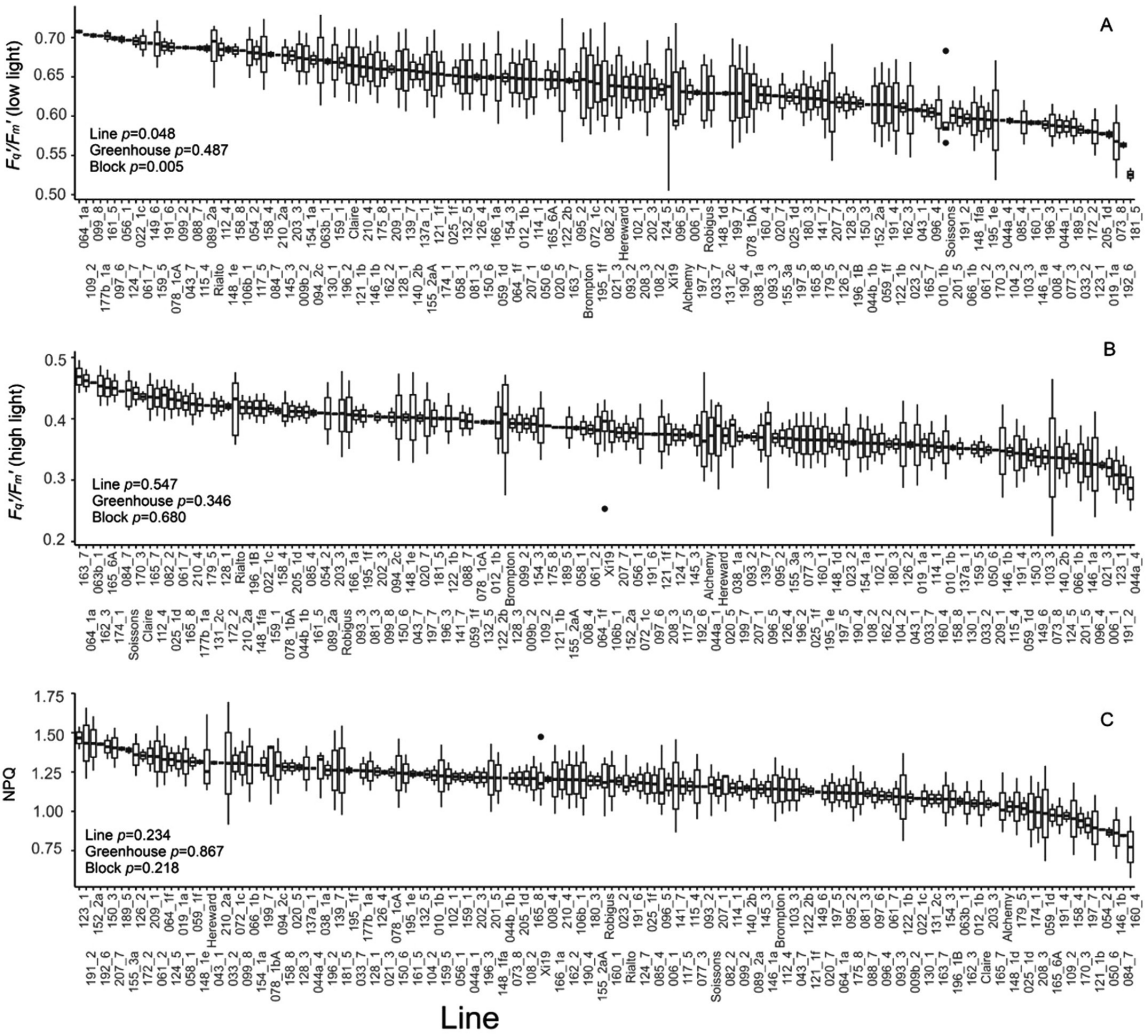
PSII operating efficiency at 100 µmol m^–2^ s^–1^ PPFD (*F*_q_'*/F*_m_' low light), PSII operating efficiency after 30 min at 1000 µmol m^–2^ s^–1^ PPFD (*F*_q_'*/F*_m_' high light), and non-photochemical quenching after 30 min at 1000 µmol m^–2^ s^–1^ PPFD (NPQ). Data were estimated via chlorophyll fluorescence imaging. For graphs, horizontal lines within boxes indicate the median and boxes indicate the upper (75%) and lower (25%) quartiles. Whiskers indicate the ranges of the minimum and maximum values. Data were analysed with ANCOVA (*n*=2–10), and *P*-values are shown in the graph.

The operating efficiency of PSII (*F*_q_'/*F*_m_') at low light levels showed significant variation between lines (*P*=0.048), with an average value of 0.62 (Fig. 5A). In the parental lines, the lowest operating efficiency was observed in Soissons while Rialto displayed the highest value (0.68). When plants were exposed to the subsaturating light intensity of 1000 µmol m^–2^ s^–1^, operating efficiency decreased and no significant differences were observed between lines. Similarly, although variation was present for NPQ, this was not significantly different between lines, potentially due to the subsaturating light conditions to which the plants were exposed.

### Stomatal density

**Fig. 6.**
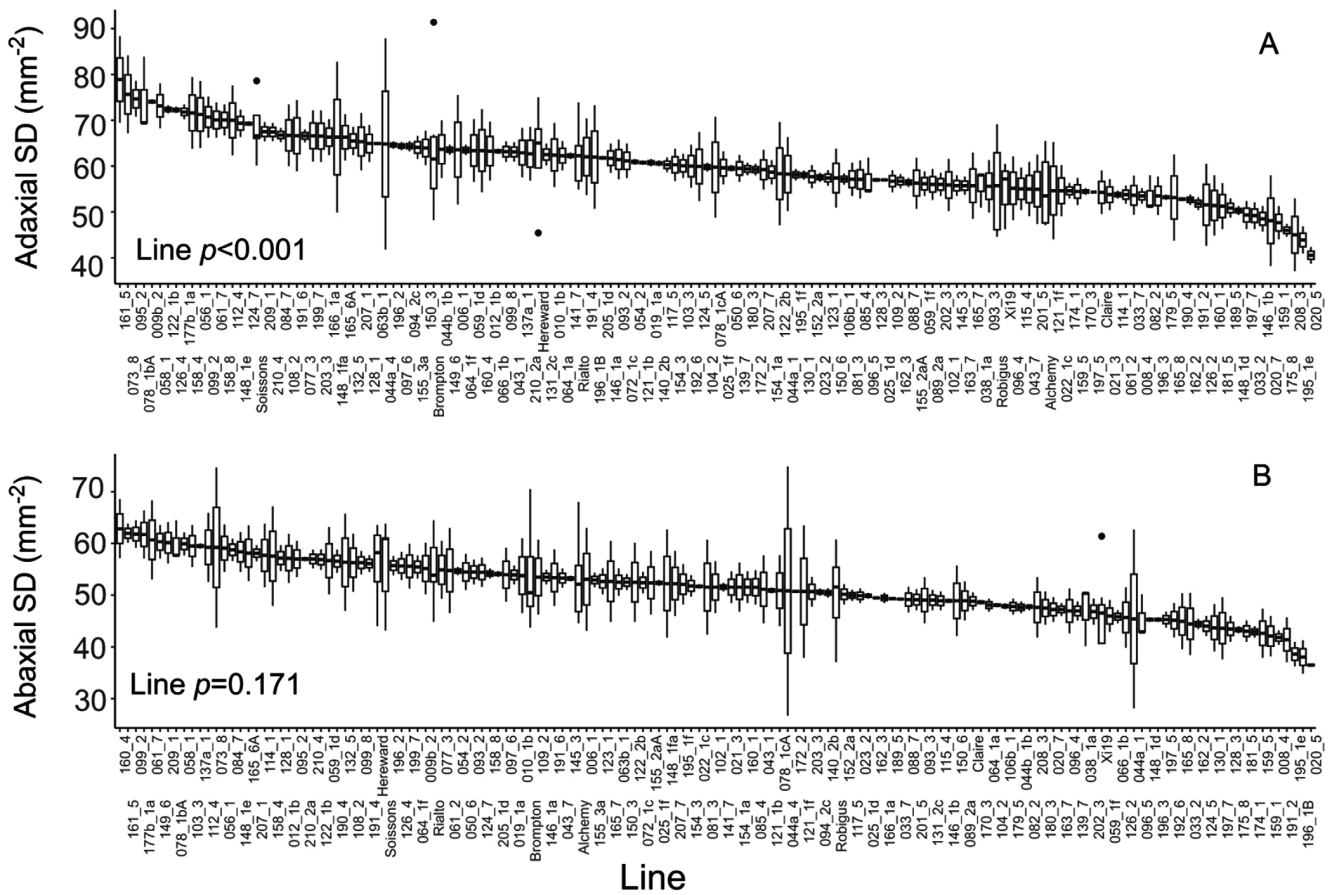
Stomatal anatomical traits for a subset of the lines used for photosynthetic and dynamic screening. In (A), the adaxial stomatal density (SD) is shown while in (B) the abaxial SD is presented. For graphs, horizontal lines within boxes indicate the median and boxes indicate the upper (75%) and lower (25%) quartiles. Whiskers indicate the ranges of the minimum and maximum values. Data were analysed with one-way ANOVA (*n*=2–10), and *P*-values are shown in the graph.

**Fig. 7.**
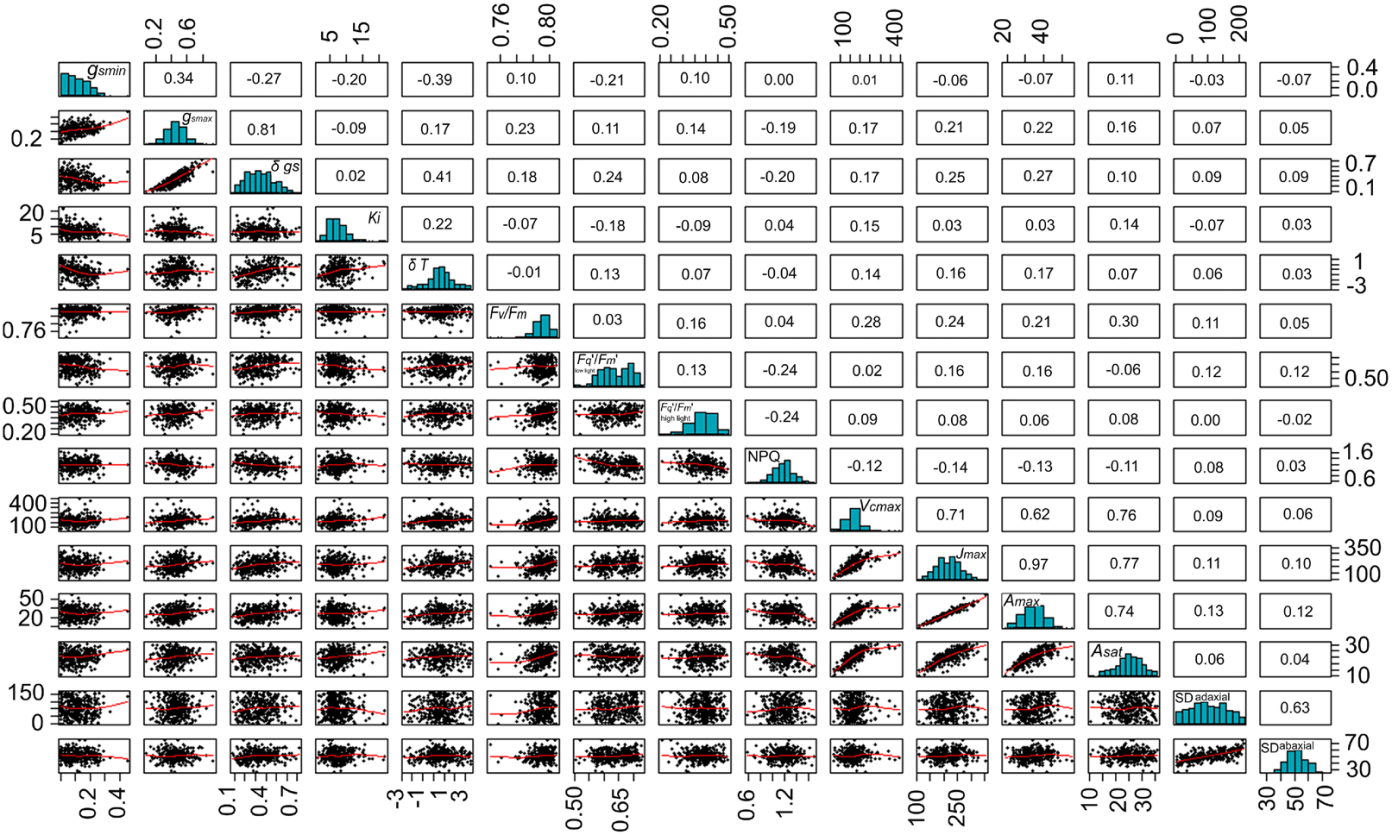
Multiple scatter plots for traits assessed in this work. In each panel, the Pearson coefficient is shown for each correlation as well as the distribution. Significant associations are discussed in the text.

Adaxial stomatal density was generally greater than abaxial density in all lines, which is typical for wheat although uncommon in most other species (Wall *et al.*, 2022). Adaxial stomatal density showed significant (*P*<0.05, Fig. 6A) variation between lines analysed, with average stomatal density values ranging between 50 stomata mm^–2^ and 80 stomata mm^–2^, while average abaxial stomatal density values were between 40 stomata mm^–2^ and 65 stomata mm^2^. Alchemy and Soissons had the highest average stomatal densities (57 stomata mm^–2^ and 61 stomata mm^–2^, respectively) of the parental lines while Claire had the lowest (50 stomata mm^–2^). There was a strong positive correlation (*P*<0.001) between adaxial and abaxial stomatal density (Fig. 7), suggesting a link between the two surfaces in terms of cell differentiation into guard cells.

Correlation analysis including all measurement parameters showed a number of significant associations (Fig. 7). Significant correlations (*P*<0.05) were observed between *A*_sat_, *A*_max_, *V*_cmax_, and *J*_max_. No significant associations were observed between stomatal anatomical features and estimated dynamic or steady-state *g*_s_ traits. However, a negative and significant correlation was observed between *g*_smin_ and *K*_i_ (*P*<0.001). Similarly, δ*T* significantly and positively correlated with *K*_i_ while a negative association existed between δ*T* and *g*_smin_. δ*g*_s_ was correlated with several traits (*J*_max_ and *A*_max_) and, in particular, with δ*T* (*r*=0.47).

## Discussion

The need to double food production in the next 50 years to feed a growing human population (Ray *et al.*, 2019; Asseng *et al.*, 2020; Furbank *et al.*, 2020; Billen *et al.*, 2024) and the requirement to do so in the face of predicted climate changes has led to increased research efforts to improve photosynthesis and other physiological processes in crops. Genetic modification (GM) of photosynthetic pathways has already proven successful (Raines, 2011; Evans, 2013; Kromdijk *et al.*, 2016; South *et al.*, 2019; Voss-Fels *et al.*, 2019); however, GM production is still met with resistance in many countries, and therefore exploiting the natural variation that exists in key crop characteristics represents an exciting and unexploited alternative.

### Natural variation in photosynthetic capacity

Photosynthetic traits have been recognized previously as a potential source of natural variation that could be exploited for incorporation into breeding programmes to increase yield (Lawson *et al.*, 2012; Driever *et al*., 2014; Faralli *et al*., 2019a). Using standard IRGA techniques (i.e. *A/C*_i_) allowed us to take photosynthetic measurements in nearly 200 wheat genotypes from a germplasm that captures 80% of the single nucleotide polymorphism variation in North-West European bread wheat (Mackay *et al*., 2014), providing the largest screen to date of several photosynthetic parameters of the flag leaf. We observed significant variation in both photosynthetic capacity (*V*_cmax_ and *J*_max_Fig. 3, and *A*_max_Fig. 2) and light-saturated rates of photosynthesis (*A*_sat_), highlighting the extent of intraspecific diversity that exists within UK bread wheat. The strong correlation we detected between *V*_cmax_ and *J*_max_ (Fig. 7) supports observations reported by others (Wullschleger, 1993). Furthermore, a simulation analysis in rice suggested that genetic variation in both Rubisco-limited (*V*_cmax_) and electron transport-limited (*J*_max_) photosynthesis increased rice yields by 22–29% across distinct locations and years (Yin and Struick, 2017), potentially providing genetic targets for exploitation to improve wheat photosynthesis in a similar way.

Although questions have been raised regarding how closely linked photosynthetic rates per unit of leaf area are with yield (Driever *et al.*, 2014; Zanella *et al.*, 2023), ultimately photosynthesis is the primary determinant of all plant metabolic processes and therefore inevitably associated with reproductive processes (Slafer and Araus, 2007) and yield (Long *et al*., 2006; Parry *et al.*, 2011). Furthermore, many studies have reported increases in photosynthetic CO_2_ assimilation along with rises in yield when different C_3_ crops were grown in FACE facilities (Ainsworth and Long, 2005). Similarly, significant positive associations have been observed for different breeding lines in the field between CO_2_ uptake and *g*_s_ with grain yield components (Fischer *et al.*, 1981, 1998; Blum, 1990; Reynolds, 2000; Carmo-Silva *et al*., 2017). However, contrary to this, many studies that have reported considerable variation in photosynthesis could not demonstrate that these differences translated into changes in yield (e.g. Chytyk *et al.*, 2011; Sadras *et al*., 2012; Driever *et al*., 2014). These contradictory findings may at least partially be due to the method used for photosynthetic assessment (Driever *et al*., 2014). Indeed, in most of the studies focusing on exploiting natural variation, photosynthesis is measured as maximum capacity which does not represent photosynthetic rates achieved in the field (Lawson *et al.*, 2012). Although instantaneous measurements during the day and standardized for a specific leaf (Gaju *et al.*, 2016) provide a ‘real’ measure of photosynthesis at a specific moment in time, they fail to account for differences in microclimates, diurnal intracanopy variation, conditional effects prior to measurement, or circadian influences, all of which influence the measurements taken (Lawson *et al.*, 2012; Driever *et al.*, 2014; Faralli *et al*., 2019a). Here, we also determined the light-saturated rate of photosynthesis (*A*_sat_) (from the *A*/*C*_i_ analysis) which could be achieved in the field with sufficient light and no diffusional constraints from stomata (Lawson and Morison, 2004).

Significant associations were observed between *A*_sat_ and *V*_cmax_/*J*_max_, suggesting that measuring *A*_sat_ (Fig. 7) could provide an important step to further define potential methods for estimating the maximum carboxylation capacity of Rubisco and maximum electron transport for RuBP regeneration in wheat. Such strategies could be extremely beneficial for exploring and exploiting natural variation in photosynthesis as this is currently limited by the lengthy process for *A*/*C*_i_ measurements. While significant advances have been made recently in remote high-throughput measurements of photosynthetic capacity through hyperspectral imaging (Meacham-Hensold *et al.*, 2020; Burnett *et al.*, 2021), further research is needed to enhance the predictive capabilities of these tools across various crops and environmental conditions.

Although there was strong correlation between *A*_sat_ and *A*_max_, the differential rankings emphasize the influence of other factors on photosynthesis, for example limitation by low *g*_s_ which could impose a diffusional constraint on *A*_sat_ that would be removed by the high [CO_2_] used to measure *A*_max_. *A*_sat_ is very likely to represent realized carbon assimilation in the field, further emphasizing that a high photosynthetic potential does not always translate to a higher realized *A* as dynamic conditions and the impact on physiological processes need to be taken into consideration (see below).

The strong coordination between *J*_max_ and *V*_cmax_ both within and between species that has been demonstrated here and in other studies (Wullschleger, 1993), including those in which photosynthesis has been manipulated (Harrison *et al*., 2001), suggests that plants employ a conservative strategy (Wullschleger, 1993). It has been suggested that this is to reduce the possibility of photoinhibition when carboxylation is limited; however, this could also limit the maximum photosynthetic rate under low light intensity (Walker *et al*., 2014). In our work, the lack of any relationship between *V*_cmax_ and *J*_max_ with stomatal anatomy and/or rapidity suggests that operational *g*_s_ may limit photosynthetic carbon gain in wheat, corroborating the hypothesis in which speedy stomata may be a preferable trait for maintaining or increasing carbon fixation under a natural environment (Lawson and Blatt, 2014).

### Natural variation in dynamic stomatal responses

In order to function efficiently, CO_2_ uptake for photosynthesis must be balanced with water loss from the plant, to ensure sufficient substrate for photosynthesis, without compromise to plant water status. Under steady-state conditions, there is usually a strong correlation between *g*_s_ and *A* (Wong *et al.*, 1979); however, under dynamic conditions as experienced in the field, the slow stomatal response times to changing environmental conditions such as dynamic light patterns lead to a disconnect between *g*_s_ and *A* (Lawson and Vialet-Chabrand, 2018). The relatively slower change in stomata compared with changes in photosynthetic rate can lead to diffusion constraints limiting rates of carbon assimilation (Lawson and Blatt, 2014; McAusland *et al.*, 2016; Vialet-Chabrand, *et al.*, 2017; Lawson and Vialet-Chabrand, 2018). Therefore, the kinetics of *g*_s_ responses to changing light intensity have been identified as a potential novel trait to increase photosynthesis (Long *et al.*, 2022) and plant water use efficiency (Lawson *et al.*, 2010; Lawson and Blatt, 2014; Matthews *et al.*, 2017). Previous work on natural variation for dynamic stomatal responses in wheat has been limited by the throughput, and therefore has been restricted to the evaluation of only a handful of genotypes. This is because such measurements usually rely on the use of IRGAs to measure stomatal responses during a step change in irradiance (e.g. McAusland *et al.*, 2016) which limits the throughput of this approach. Here, we used a novel thermal screening method to examine stomatal kinetics in ca. 200 wheat genotypes. Leaf temperature is ultimately determined by stomatal conductance (as well as boundary layer and other physical features), and therefore thermography can be used to determine differences in *g*_s_ (Jones *et al*., 2009) and has been successfully employed as a screen for identifying stomatal mutants (Wang *et al*., 2004). It has also been proven to provide a rapid screen for *g*_s_ kinetics in response to changing irradiance (Vialet-Chabrand and Lawson, 2019, 2020; Kimura *et al.*, 2020; Yamori *et al.*, 2020), although this requires a range of references for energy balance calculations (see Vialet-Chabrand and Lawson, 2019).

In order to use thermography to evaluate changes in *g*_s_ between different plant specimens and/or under changing conditions, temperature references (e.g. wet and dry reference) need to be included and maintained throughout the imaging process (see Vialet-Chabrand and Lawson, 2019, 2020). Additionally, knowledge of the boundary layer conductance and considerable computation power are required. Here we used a simplified approach that employed only the dry reference (Leinonen *et al*., 2006) along with a controlled-environmental chamber (similar to the one described by McAusland *et al.*, 2013 but modified for use in grasses) that controlled and maintained conditions around the leaves for image capture. The use of the chamber creating a constant boundary layer (see Supplementary Dataset S1 for the calculation of boundary layer) simplified the calculation of *g*_s_ from thermal signatures. To validate the use of thermal images to determine the rapidity of *g*_s_ responses (*K*_i_) to a step increase in light (Fig. 4), we demonstrated a strong and significant positive correlation between *K*_i_ using both methods, and no significant differences between individual genotypes were observed. However, a greater variation in *K*_i_ from IRGA measurements was evident, suggesting that *K*_i_ from thermography may if anything underestimate *K*_i_. This can most likely be explained by the fact that thermal images take account of the spatial and temporal variation across the leaf surfaces that is not required with the smaller chambers of IRGAs, and therefore possibly provide a more realistic representation of whole-leaf responses.

As with the photosynthetic parameters, we demonstrated significant variation in both steady-state *g*_s_ at 100 µmol m^–2^ s^–1^ PPFD and the speed of *g*_s_ responses (*K*_i_).

In general, natural variation was previously detected in these traits, and this intraspecific variation was associated with stomatal anatomy in both wheat and barley (Faralli *et al*., 2019a; Stevens *et al*., 2021) in a few genotypes. Although hypothesized and observed in studies focusing on interspecific diversity (e.g. Drake *et al.*, 2013), in the present work no link was found between stomatal anatomy and rapidity. Several anatomical, structural, and biochemical factors may affect stomatal rapidity to changing light intensity, and our data suggest that processes related to signalling, osmoregulation, or solute transport play a greater role than anatomical features alone (Lawson and Vialet-Chabrand, 2018) in the variation observed. Significant associations were also observed between evaporative cooling and the magnitude of stomatal opening (δ*g*_s_), suggesting, not surprisingly, that the degree of stomatal opening is a key component for maintaining optimal leaf temperature under dynamic irradiance. Indeed, previous studies have associated steady-state maximum stomatal conductance with yield and canopy temperature depression under water-limiting and high temperature conditions (Fischer and Stockman, 1986), suggesting that both stomatal dynamics and steady-state *g*_s_ represent a key target for maintaining optimal leaf temperature for photosynthesis. The fact that *K*_i_ and δ*g*_s_ were correlated with *g*_smin_ suggests that maintaining high basal *g*_s_ under low light could represent an important trait that primes a faster stomatal responsiveness to changes in light intensity_._ In the genetic material studied here, *g*_smin_ and *g*_smax_ were positively associated, and this may indicate that potential combinations of preferable traits (e.g. inherently high *g*_smax_, low *K*_i_, and high evaporative cooling) are present in wheat and that this can be exploited for additional fine-tuning of gas exchange dynamics under fluctuating conditions. Wheat is amphistomatous and atypical, having greater stomatal numbers on the adaxial surface (Fig. 6), although this is often correlated with the abaxial density, as shown here, which suggests a common signal that determines cell differentiation between the two surfaces. Furthermore, previous studies have shown that *g*_s_ is generally higher on the adaxial surface, which could have implications for gaseous diffusion and evaporative cooling for maintaining photosynthesis (Wall *et al.*, 2022). This also highlights the potential of understanding the genetic targets that control stomatal development on the two surfaces to exploit for plants with enhanced diffusional capacity and/or cooling capacity and produce idiotypes for specific environments.

Interestingly, desirable stomatal traits were not necessarily observed in the same varieties as the desirable photosynthetic traits, suggesting that the presence of some traits may be at the expense of others. For example, the parental line Hereward had one of the fastest stomatal kinetic responses, but also one of the lowest *A*_sat_ and *V*_cmax_ values, whilst varieties such a Xi19 which had one of the highest photosynthetic capacities had one of the lowest *K*_i_ values. The rapid *g*_s_ responses in Hereward did not, however, result in a high δ*T*, illustrating the importance of measuring actual *g*_s_ values and not just rapidity alone (Lawson and Blatt, 2014). However, as expected, *g*_s_ could explain some of the photosynthetic responses observed. The parental line Robigous had low *g*_smax_ values, which most probably provides an explanation for the low *A*_sat_ observed, despite *A*_max_ being somewhere in the middle of the range. The low *g*_s_ values most probably created a diffusional constraint preventing high *A*_sat_ values from being achieved, which was overcome with the high CO_2_ for the *A*_max_ measurements.

The dual imaging system used here for the kinetic responses incorporated chlorophyll fluorescence imaging of photosynthetic efficiency (*F*_q_'*/F*_m_') (Fig. 5) alongside thermography. Cultivar differences in *F*_q_'*/F*_m_' were apparent at the lower light intensities, reflecting the variation observed using IRGA measurements; however no significant differences at the high light intensity were found. This is most probably explained by the fact that firstly, *F*_q_'*/F*_m_' decreases with illumination and therefore the values are greatly reduced, hence limiting the potential variation range; and secondly in C_3_ species, that the end-products of electron transport (ATP and NADPH) can also be utilized by sinks other than CO_2_ assimilation (such as photorespiration) (Baker, 2008). For this reason, the measurements of *F*_q_'*/F*_m_' are unable to distinguish differences in *A* that can be achieved through gas exchange, including those as a result of *g*_s_ diffusional constraints.

### Conclusion

This is the first study providing evidence of wide variation for steady-state and dynamic gas exchange traits in a bread wheat MAGIC population. This variation was detected using both standard eco-physiological approaches (IRGA) and novel methods (thermal imaging), allowing high throughput for stomatal dynamic phenotyping. Since natural variation in photosynthetic traits was identified in the wheat genotypes investigated, further work should focus on detecting the genetic loci controlling the traits employing larger numbers of RILs. Similarly, variation in dynamic stomatal responses was observed for the first time in the population as well as for *g*_smax_, Δ*g*_s_, and evaporative cooling using this novel high throughput method. This provides evidence of variability in bread wheat for dynamic *g*_s_ traits potentially providing unexploited targets for incorporation into ongoing breeding programmes. The strong relationships between our measured traits also provide proof of concept that taking the simpler measurement of *A*_sat_ and/or *A*_max_ could serve as a proxy for the more complex and time-consuming biochemical measurements of photosynthetic potential *V*_cmax_ and *J*_max_. This is the first phenotyping study illustrating evidence of wide phenotypic variation in a wheat experimental population for several key traits important in yield determination, thus stressing the possibility to further exploit this variation for detecting the genetic control of stomatal and photosynthetic characters for crop improvement.

## Supplementary data

The following supplementary data are available at *JXB* online.

Fig. S1. Visualization of the pipeline for plant growth including vernalization and subsequent greenhouse growth

Dataset S1. Spreadsheet for calculating boundary layer conductance.

Dataset S2. Spreadsheet for calculating *g*_s_ from thermal measurements.

erae233_suppl_Supplementary_Figure_S1

erae233_suppl_Supplementary_Datasets_S1-S2

## Data Availability

Raw data can be accessed from the Dryad Digital Repository (Faralli *et al.*, 2024) (https://doi.org/10.5061/dryad.79cnp5j4d).
