# Supplementary material for: Exploring natural genetic diversity in a bread wheat multi-founder population: dual imaging of photosynthesis and stomatal kinetics
Source: J Exp Bot. 2024 May 25;75(21):6733–47. doi: 10.1093/jxb/erae233 (PMC11565207; doi:10.1093/jxb/erae233)
Supplement: erae233_suppl_Supplementary_Figure_S1 [file erae233_suppl_supplementary_figure_s1.pdf]

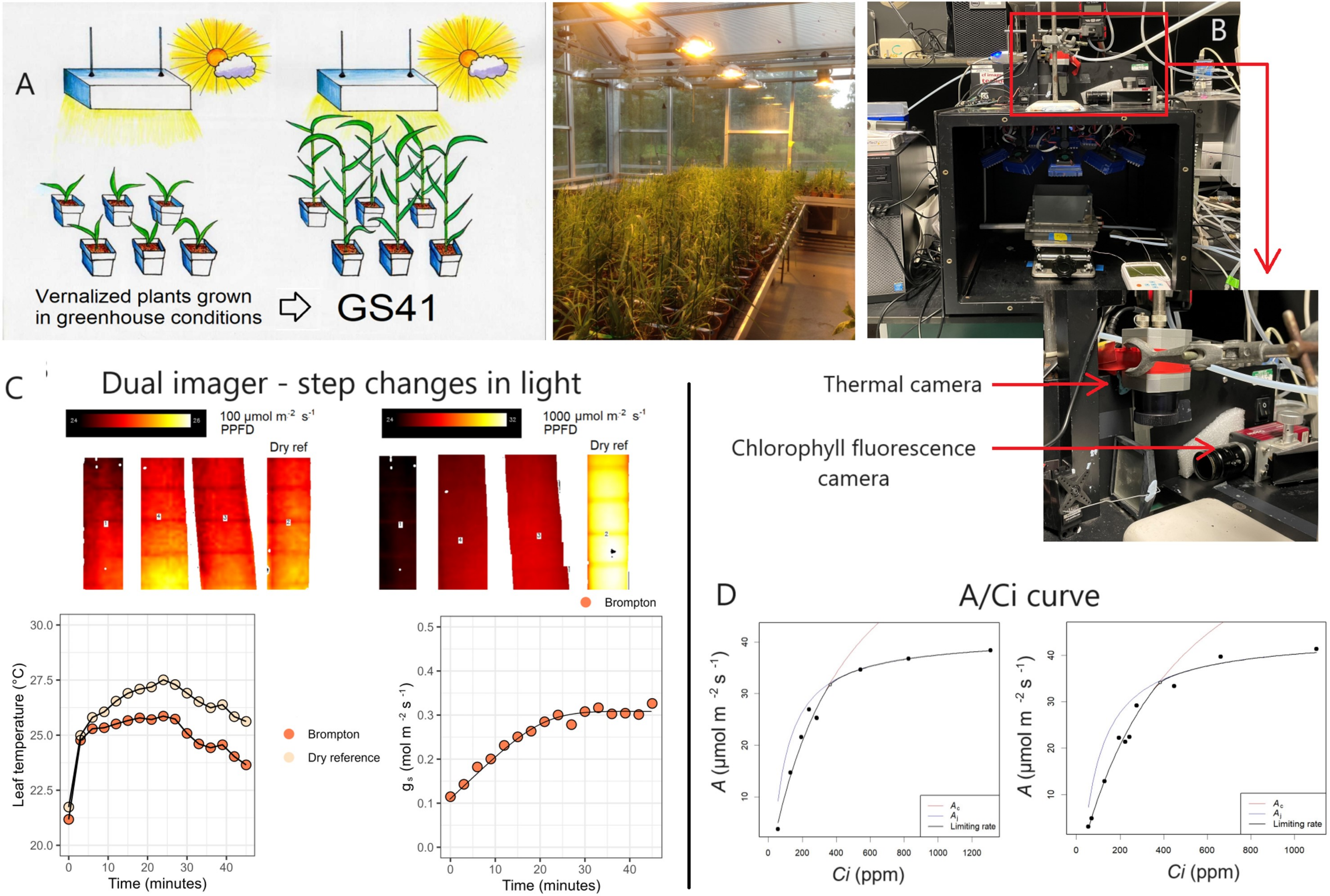

Supplementary Figure 1. A) Visualization of the pipeline for plant growth including vernalization and subsequent greenhouse growth. B) Dual imager set-up including thermal and chlorophyll fluorescence cameras as well as the cuvette within the system used for leaf screening. C) Example of thermal images and spatial variation assessed including leaf (cv. Soissons) and dry reference with relative example of temperature and g_s dynamics evaluated. D) Example of contrasting A/C_i curves and relative standard output. A_n is the net photosynthetic rate, *Ci* the intercellular CO_2 concentration. Symbols are measurements, the black line the fitted model. Coloured lines indicate the two photosynthesis rates for Vc_{max} and J_{max} estimation,
